# SAFARI: shape analysis for AI-segmented images

**DOI:** 10.1186/s12880-022-00849-8

**Published:** 2022-07-22

**Authors:** Esteban Fernández, Shengjie Yang, Sy Han Chiou, Chul Moon, Cong Zhang, Bo Yao, Guanghua Xiao, Qiwei Li

**Affiliations:** 1 Department of Mathematical Sciences, The University of Texas at Dallas, Richardson, TX USA; 2 Quantitative Biomedical Research Center, Department of Population and Data Sciences, The University of Texas Southwestern Medical Center, Dallas, TX USA; 3 Department of Statistical Science, Southern Methodist University, Dallas, TX USA

**Keywords:** Medical imaging, Machine learning, Shape representations, Shape descriptors

## Abstract

**Background:**

Recent developments to segment and characterize the regions of interest (ROI) within medical images have led to promising shape analysis studies. However, the procedures to analyze the ROI are arbitrary and vary by study. A tool to translate the ROI to analyzable shape representations and features is greatly needed.

**Results:**

We developed SAFARI (shape analysis for AI-segmented images), an open-source R package with a user-friendly online tool kit for ROI labelling and shape feature extraction of segmented maps, provided by AI-algorithms or manual segmentation. We demonstrated that half of the shape features extracted by SAFARI were significantly associated with survival outcomes in a case study on 143 consecutive patients with stage I–IV lung cancer and another case study on 61 glioblastoma patients.

**Conclusions:**

SAFARI is an efficient and easy-to-use toolkit for segmenting and analyzing ROI in medical images. It can be downloaded from the comprehensive R archive network (CRAN) and accessed at https://lce.biohpc.swmed.edu/safari/.

**Supplementary Information:**

The online version contains supplementary material available at 10.1186/s12880-022-00849-8.

## Background

Medical images are produced from different modalities such as X-ray, computational tomography (CT), magnetic resonance imaging (MRI), whole-slide imaging (WSI). These procedures produce massive imaging data, which capture the anatomy and physiological processes of the body or histological details in high spatial resolution. Recent developments in deep-learning methods have enabled the automatic detection of regions of interest (ROI), such as tumor regions, in medical images [1]. These newly developed methods and other standard image processing algorithms produce pixel-based representations of the medical images, known as artificial intelligence (AI)-segmented images. These segmented images facilitate the identification and analysis of the ROI within the raw images.

Analyses of these ROIs can produce clinically meaningful information that characterizes conditions or diseases and predict patient outcomes. Multiple studies in brain, breast, and lung cancer have used tumor shape to predict patient prognosis [1–7]. A recent study in lung cancer used digital hematoxylin and eosin (H &E)-stained pathology images to associate certain shape characteristics with patient survival outcomes [1]. These studies generally rely on shape features such as boundary descriptors [2, 3], geometric descriptors [1], landmark-based descriptors [7], and topological summaries [6, 8]. Such shape features are computed by various shape representations that characterize the ROI in one or two dimensions. For the shape features to be meaningful, they should (1) quantify the shape, geometry, and topology of the regions; (2) be translation, rotation, and scale-invariant; and (3) be application-dependent with a low computational complexity [9, 10].

**Fig. 1:**
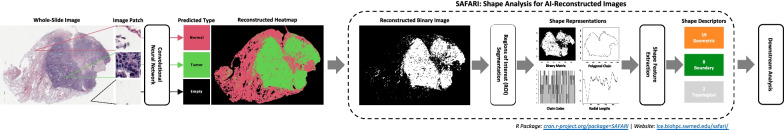
SAFARI package workflow: (1) whole-slide image is processed by an Automated Tumor Recognition System (ATRS) and converted into a binary format, (2) ROI are identified and segmentedfrom the input binary image, (3) shape features are simultaneously computed for the downstream analysis

While these studies have relied on raw or segmented images, the data processing and quality control steps are usually arbitrary, study-dependent, and rely on some software tool or programming script. As a result, there lacks an open-source implementation that can translate different shape representations, extract quantitative shape features from the ROI, and summarize the results. To meet the increasing demand for such a tool, we developed an open-source R package, SAFARI (Shape Analysis for AI-segmented Images), for ROI labelling, representation, feature extraction, and visualization. These procedures and the preliminary steps to prepare images for SAFARI are shown in Fig. 1. Additionally, we provide a user-friendly online analytic tool.

## Implementation

**Fig. 2:**
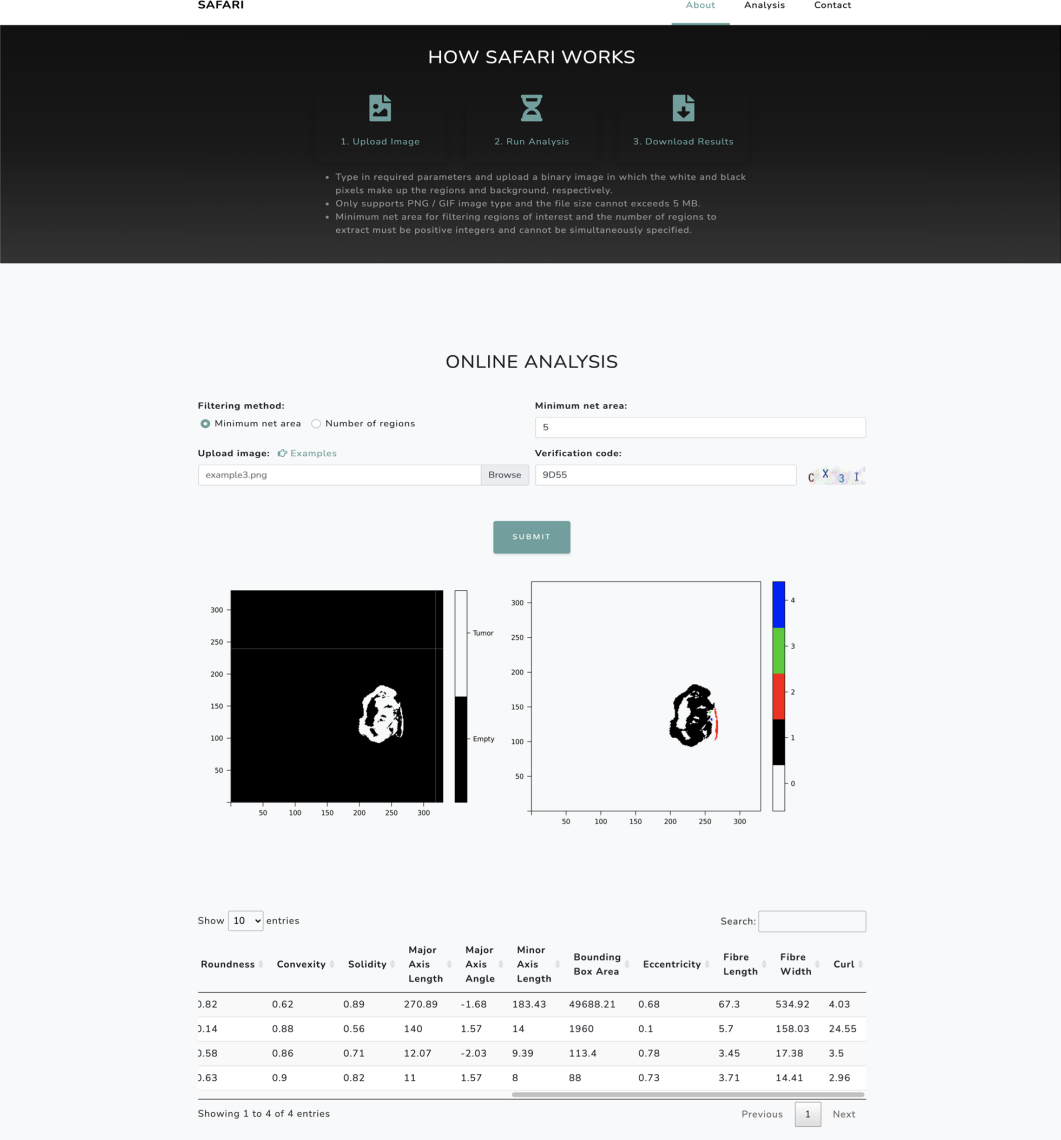
SAFARI online analysis interface, instructions, and results page

SAFARI is an open-source R package with a user-friendly online tool. The graphical interface offers a demonstration of the package’s capabilities. Given a valid segmented image, SAFARI can automatically detect, segment, and quantify the ROI. The main deliverables of the SAFARI package are listed in Additional file 1: Table S1. When using the online tool, the resulting segments will be displayed on the website alongside a table corresponding to the shape features of each segment (Fig. 2). While the current version of our R package supports up to three-class segmented images, the online tool only accepts binary images (PNG/GIF $<\,3$ MB). The latest development and released versions of the R package are available on GitHub [11] and CRAN [12], respectively.

We implemented a processing procedure to (1) segment the ROI from an AI-segmented image, (2) translate to different shape representations, and (3) extract a variety of shape features based on those representations. The development of this pipeline is motivated by the “AI-segmented image” case study in [1], but we note that our tool can be used for the analysis of any binary and three-class image.

### ROI labelling

Standard image processing methods, including newly-developed deep-learning techniques [1], generate pixel-based image representations that are easy to manipulate, process, and store. These methods map the regions within the image to integer codings, referred to as categories. In X-rays, these categories represent the empty and skeletal structures. In pathology images, they represent the empty, malignant, and non-malignant regions. We show an example of an H &E-stained pathology image, converted to a three-class segmented image in Additional file 1: Fig. S1. We can easily identify ROI made up of these categories through this process, such as tumors tissues.

Individual ROI are identified and segmentedby standard morphological operations, based on a 4-connectivity [13]. To reduce the influence of smaller regions, two filtering methods are available based on a user specifying a minimum net area or the largest *n* regions to keep. The resulting ROI, stored in a single integer matrix, are labeled from largest to smallest in area.

### Shape representations

Shape objects are then created from the segmentedROI; specifically, for each region, a binary matrix that indicates the object and a polygonal chain of its boundary (see an example in Additional file 1: Fig. S2). We can derive further shape representations, such as the (normalized) radial lengths and (curvature) chain codes from the polygonal chain. These are one-dimensional and are able to quantify the contour and directional changes in the boundary. Additional properties can be computed from the polygonal chain, which are the convex hull and minimum bounding box. For more details, regarding the six primary and derived shape objects, see Additional file 1: Section S1 and Table S2.

### Feature extraction

The resulting shape objects provide a heterogeneous feature extraction that is able to quantify different information about the ROI. Various measurements that quantify the shape, geometry, and topology of the regions are computed, and categorized as geometric, boundary, and topological shape features. About 30 shape features, properly categorized, are shown in Table 1 and detailed in Additional file 1: Table S3 by their formulae and properties. The dependencies between shape representations and features are shown in Additional file 1: Fig. S3, respectively. These region-level shape features can be used in supervised and unsupervised applications. More importantly, they can further serve to characterize patients and the underlying condition or disease of interest.

**Table 1:** Overview of the 29 shape features in three categories

Category	Features
Geometric	Net area, thickness, elongation, filled area, perimeter, circularity, fibre length, fibre width, convex area, convex perimeter, roundness, convexity, solidity, major axis length, major axis angle, minor axis length, bounding box area, eccentricity, and curl
Boundary	Bending energy, total absolute curvature, radial mean, radial standard deviation, entropy, area ratio, zero crossing count, and normalized moment classifier
Topological	Number of holes and number of protrusions

For a full table and a diagram, refer to Additional file 1: Table S3 and Fig. S3, respectively

## Results

We studied the relationship between tumor shape and survival outcomes in lung and brain cancer patients, extending the work in [1] and following a similar methodological approach as in [8], respectively. All analyses were performed with the R software, version 4.0.3, and R packages survival (version 3.2-7) and glmnet (version 4.1) [14–16].

### Dataset A

We used 246 pathology images from 143 consecutive patients with stage I–IV non-small-cell lung cancer in the National Lung Screening Trial (NLST) [17]. The patient characteristics are summarized in Additional file 1: Table S4. All patients had undergone surgical procedures as treatment. The survival time was defined as the period from the time of the surgery until death or the final date of the study (December 31, 2009). Forty-five patients had died during this time period, and the remaining 98 were still alive at the final date of the study. As a result, the survival time of the alive patients was censored. There were multiple tissue slides scanned at $40\times$ magnification for each patient. The median size of the slides was $24,244 \times 19, 261$ pixels. Based on a convolutional neural network, the automated tumor recognition system developed by [1] created a segmentedthree-class image of each slide. A binary version of the three-class image was created, where the holes within the tumors represent the empty and non-malignant regions.

### Downstream analysis I: association study

Before starting the downstream analyses, we first implemented a quality control step. Any ROIs with a net area less than one-fourth of the largest ROI of each slide were removed. The 29 tumor-level features extracted by SAFARI were then average at the slide level.

To investigate the association with overall survival, we fit a separate univariate Cox proportional-hazards (CoxPH) model to each shape feature at the slide level. We summarize the results in Additional file 1: Table S5, where the shape features were centered and scaled, and patients with multiple slide images were accounted for by clustering. Notably, 14 of the 29 features were statistically significant (*p* value $\le$ 0.05). Out of the 14 features, 12 were geometric, and two were topological. Additionally, all significant features had negative effects to a poor survival outcome (hazard ratio $>\, 1$). Finally, the major axis angle served as a negative control and was not statistically significant, as expected, with a *p* value of approximately 0.86 (Additional file 1: Table S5). Since our methodology is an extension to [1], we compare their results to ours where 4 shape features were statistically significant (*p* value $\le$ 0.05) and not included in the original study (Additional file 1: Table S6).

### Downstream analysis II: predictive performance

**Fig. 3:**
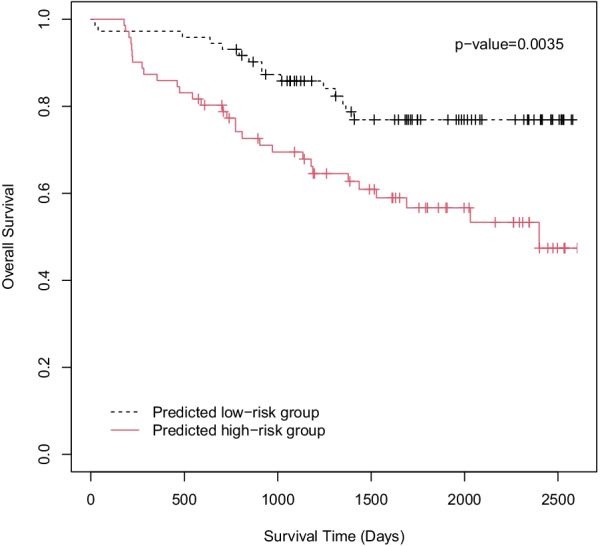
Survival curves, estimated using the Kaplan-Meier method, for both high-risk and low-risk groups

We choose a small subset of features by fitting a regularized CoxPH model with a LASSO penalty to prevent overfitting. The tuning parameter $\lambda$ was selected by ten-fold cross-validation [1, 15]. The selected features were the major axis length, circularity, and eccentricity. We show the cross-validation results and the importance of each selected feature in Additional file 1: Fig. S4. To evaluate the prognostic performance of the selected shape features, we predicted the risk scores using leave-one-out cross-validation. Within each cross-validation fold, a single sample was chosen where we predicted its risk score by training a CoxPH model on the remaining 245 samples. The predicted risk scores, based on the relative risk of the fitted models, were averaged for each patient. Subsequently, the patients were dichotomized into high and low-risk groups, using the median patient-wise risk score and resulting in two groups with 71 and 72 samples, respectively. A Kaplan–Meier plot of the high and low-risk groups is shown in Fig. 3. The *p* value of the log-rank test was 0.0035, demonstrating a separation between the two groups. Additionally, the prognostic performance of the shape-based risk scores was validated by a multivariable CoxPH model. After adjusting for clinical variables, including age, gender, smoking status, and stage, the predicted risk groups independently predicted prognosis (high-risk vs. low-risk, hazard ratio = 2.32, *p* value = 0.0134, see Table 2).

**Table 2:** Multivariable analysis of the predicted risk group

	Hazard Ratio (HR) with $95\%$ confidence interval (CI)	*p* value*
High-risk versus low-risk	2.32 (1.19–4.52)	**0.0134**
Age	1.09 (1.03–1.16)	**0.0040**
Male versus female	0.92 (0.49-1.74)	0.7964
Smoker versus non-smoker	0.94 (0.51–1.72)	0.8429
Stage II versus stage I	1.30 (0.44–3.88)	0.6323
Stage III versus stage I	3.79 (1.93–7.45)	≤ **0.001**
Stage IV versus stage I	4.26 (1.67–10.83)	**0.0024**

A Cox proportional-hazards (CoxPH) model was fitted to test the predictive performance of the predicted risk score, adjusted for clinical variables and based on the leave-one-out cross-validation results

*Bolding signifies features with *p* value ≤ 0.05.

### Dataset B

We used the MRI scans of 61 patients with Glioblastoma (GBM), the most common malignant grade IV brain tumor, obtained from The Cancer Imaging Archive (TCIA) [18, 19] and their clinical data retrieved from The Cancer Genome Atlas (TCGA) [20]. The patient characteristics are summarized in Additional file 1: Table S4. All MRI images were segmented into tumor and non-tumor regions using the Medical Imaging Interaction Toolkit (MITK) with augmented tools for segmentation [21]. The size of the scans are either $256 \times 256$ or $512 \times 512$, and each patient has approximately 23–25 MRI images. We followed the data pre-processing steps in [8].

### Downstream analysis III: association study

We followed a similar procedure as in the first case study. A quality control step was first implemented, followed by investigating the association with overall survival. Since brain tumor images are represented by two-dimensional slices, some of which do not contain any region of the tumor, we chose the slice level with the largest tumor size. As a result, we obtained 29 shape features for each patient.

To investigate the association with overall survival, we fit a separate univariate Cox proportional-hazards model (CoxPH) to each shape feature at the patient level. We summarize the results in Additional file 1: Table S7, where the shape features were centered and scaled. Notably, 11 of the 29 features were statistically significant (*p* value $\le$ 0.05). Out of the 11 features, 10 were geometric, and one was topological. Additionally, all significant features had negative effects to a poor survival outcome (hazard ratio $>\, 1$).

## Discussion

The methodology used in the previous section is similar to the one used in [1], but we extend the study to highlight the capabilities of our tool. We increased and diversified the potential predictors of prognosis in lung cancer, by computing shape features on various shape representations, such as the chain codes, polygonal chain, radial lengths, etc. By clustering at the patient-level, we correct the standard errors and capture the heterogeneity of the tumors. This provides a different approach from [1] where they summarize the shape features at the patient-level, potentially, affecting the results due to outliers. We also applied our software to an additional case study. The results shown in the association study were promising. This evidence suggests that the shape features provided could work for a variety of datasets, especially if we consider the topological differences between lung and brain tumors.

Shape analysis has been widely studied and its usefulness has already been demonstrated in many different problems, such as lesion detection [22], classification [22–24], survival analysis [1, 25], and tissue segmentation [1, 7, 8], but the lack of complete shape analysis tools in the R environment motives our work. Although there are tools available in CRAN and Bioconductor, none have a full pipeline [26], support as many shape features and representations [27], or have applications to medical imaging [28] as our tool. Additional file 1: Table S8 compares a sample of the shape analysis tools available in the R environment to SAFARI.

While our proposed tool provides a complete, easy-to-use, and open-source shape analysis pipeline, it still has some limitations. First, the pipeline does not include the image segmentation step and heavily depends on the quality of the original segmentation. While the goodness of the segmentation stage will influence the final results, the contribution of our tool is its (1) diverse set of shape features to benchmark novel approaches, (2) simplicity for clinicians and pathologists, and (3) offline and online access. Additionally, we intend to integrate automatic segmentation in the future for specific applications. Second, we need to include more novel shape features such as boundary features proposed in [7] and topological features proposed in [8]. Since we incorporate standard shape features found in older literature, it would be best to adapt to new methods for quantifying the shape, boundary, and topology of shapes.

A final detail that needs to be considered is the case of multi-label segmentation outputs, which result in heatmaps for different classes that will be represented along channels. For this scenario, we encourage users to treat each channel separately, equivalent to the binary image consideration, when using our tool for ROI labelling and feature extraction. Since this output type is necessary for most AI-algorithms, we will consider adding functionality for segmented maps with multiple channels in a future update.

## Conclusion

We developed SAFARI, an open-source R package with its accompanying user-friendly online tool, to segment ROIs and characterize their shapes from AI-segmented images. Our lung cancer case study demonstrated how tumor shape features could predict patients’ survival outcomes. The results of this study provide new biomarkers for prognosis and further evidence of the underlying association between shape and disease progression. To our knowledge, SAFARI is one of the few tools in the R environment with such capabilities. We believe that this tool will facilitate the analysis of ROI in a plethora of applications and boost methodological research in shape analysis.

## Availability and requirements

- Project name: Shape analysis for AI-segmented images.
- Project home page: https://lce.biohpc.swmed.edu/safari/.
- Archived version: https://cran.r-project.org/web/packages/SAFARI/index.html and https://github.com/estfernandez/SAFARI.
- Operating system(s): Platform independent.
- Programming language: R.
- Other requirements: EBImage 4.32.0 or higher.
- License: GNU General Public License v3.0.
- Any restrictions to use by non-academics: None.

## Supplementary Information

**Additional file 1.** Supplementary tables an figures.

## Data Availability

Dataset A: The H&E-stained pathology images and patient clinical information were taken directly from the NLST web portal (https://cdas.cancer.gov/learn/nlst/home/). The access to the raw images must be applied through the website. The AI-segmented images analyzed in this study are available from the corresponding authors upon reasonable request. Dataset B: The MRI scans and patient clinical information were taken directly from the TCGA web portal (https://wiki.cancerimagingarchive.net/display/Public/TCGA-GBM). No application is needed to access the raw images. The AI-segmented images analyzed in this study are available from the corresponding author upon reasonable request.

## Abbreviations

CT: Computational tomography
MRI: Magnetic resonance imaging
WSI: Whole-slide imaging
ROI: Regions of interest
SAFARI: Shape analysis for AI-segmented images
HR: Hazard ratio
SE: Standard error
CI: Confidence interval
NLST: National lung screening trial
CNN: Convolutional neural network
CoxPH: Cox proportional-hazards model
