# Supplementary material for: SAFARI: shape analysis for AI-segmented images
Source: BMC Med Imaging. 2022 Jul 22;22:129. doi: 10.1186/s12880-022-00849-8 (PMC9308199; doi:10.1186/s12880-022-00849-8)
Supplement: Supplementary file 1 — Additional file 1. Supplementary tables an figures. [file 12880_2022_849_MOESM1_ESM.pdf]

SUPPLEMENTARY MATERIALS FOR "SAFARI: SHAPE ANALYSIS FOR AI-SEGMENTED IMAGES"

By Esteban Fernández Morales[1], Shengjie Yang[2], Sy Han Chiou[1], Chul Moon[3], Cong Zhang[1], Bo Yao[2], Guanghua Xiao[2,†], and Qiwei Li[1,*]

[1] *Department of Mathematical Sciences, The University of Texas at Dallas,* [*] *qiwei.li@utdallas.edu*

[2] *Quantitative Biology Research Center, Department of Population and Data Sciences, The University of Texas Southwestern Medical Center,* [†] *guanghua.xiao@utsouthwestern.edu*

[3] *Department of Statistical Science, Southern Methodist University*

S1. Supplement to Shape Representations.

S1.1. *Binary Matrix.* Let $\boldsymbol{M}_{W\times L}$ be a binary matrix representing an arbitrary W-by-L image, containing a 4-connected region, where the foreground and background are composed of ones and zeros, respectively. Additionally, we can represent each pixel in the image as a point in a 2-dimensional discrete plane, that is, each entry $M_{wl} \in \boldsymbol{M}$ can be denoted as a point $(l, w) \in \mathbb{N}^2$. Furthermore, to differentiate between foreground and background points, let $\mathrm{I}_R\colon \mathbb{N}^2 \to \{0, 1\}$ be the indicator function for an image matrix given by

$$\mathrm{I}_R(l, w) = \begin{cases} 1 & \text{if } (l, w) \text{ is a foreground pixel,} \\ 0 & \text{if } (l, w) \text{ is a background pixel.} \end{cases}$$

The indicator function I_R and distribution of points (l, w)'s will be used to recreate the region's contour in a two dimensional Cartesian plane, known as the polygonal chain.

S1.2. *Polygonal Chain.* The entries of the binary matrix $\boldsymbol{M}_{W\times L}$ that make up the contour of the region can be extracted by the Moore-Neighbor tracing algorithm, modified by Jacob's stopping criteria, with the 1) starting boundary point, 2) direction to traverse the boundary (clockwise or counter-clockwise), and 3) pixel connectivity (Gonzalez, Woods and Eddins, 2020). EBImage, and as a result also SAFARI, uses a 4-connectivity. Therefore, the first argument is trivial. For the starting boundary point, the point in the lowest left-most location is chosen. Specifically, let

$$S = \left\{ (l_i, w_i) \mid \mathrm{I}_R(l_i, w_i) = 1 \wedge w_i = \min_{1 \le k \le W} w_k \right\}$$

be the collection of points that make up the region and are located in the lowest y-coordinate such that

$$(l_1, w_1) = \begin{cases} S_1 & |S| = 1 \\ \min\limits_{l_1} S & \text{otherwise} \end{cases}$$

Applying the Moore-Neighbor tracing algorithm to the region matrix, results in the points that make up the boundary of the region, specifically, where the boundary begins at the lowest left-most area of the region and traverse through the boundary in a clockwise direction. From the boundary points, we can create a sequence of points, known as the closed polygonal chain, that represents the boundary of the region by creating a closed and simple polygon. We can see the binary matrix and its corresponding polygonal chain in Figure S2. Let $N = \sum_{l,w} \mathrm{I}_R(l, w)$ be the total number of points that make up the region and $\boldsymbol{P}_{(n+1)\times 2}$ be the collection of points that make up the closed polygonal chain of the region such that 1) $n \le N$ is the number of boundary points, 2) $P_{i\cdot} = (x_i, y_i) \in \mathbb{N}^2$, for $i = 1, \ldots, n+1$, and 3) $(x_1, y_1) = (x_{n+1}, y_{n+1})$. Through the polygonal chain, we can derive further two- and one-dimensional shape representations that can be used to compute specific descriptors.

S1.3. *Chain Code.* The slope of a shape's contour can be approximated by the directional changes between two consecutive boundary points. These directional changes can be encoded to, essentially, assign a number (from 0 to 7) to each possible relative direction resulting in an encoding list, each element known as the chain code, that provides a compact representation of the shape's contour (Wirth, 2004; Agu, 2014). Let $\boldsymbol{c}$ be a $1 \times n$ vector representing the chain codes of the polygonal chain where each

entry $c_i \in \mathbb{N} \cap [0, 7]$ corresponds to a direction in the 8-way split of the unit circle and determined by a series of steps.

First, we determine the angle between the vector composed of the difference between the two consecutive points and the x-axis, that is, let $\theta_i = \text{arctan2}(\boldsymbol{d}_i)$ be the resulting angle where $\boldsymbol{d}_i = P_{i\cdot} - P_{i+1\cdot}$ is the difference. Since $\theta_i \in [-\pi, \pi]$, we have to transform the angle to

$$\hat{\theta}_i = \begin{cases} \theta_i & \theta_i \geq 0, \\ \theta_i + 2\pi & \theta_i < 0, \end{cases}$$

such that $\hat{\theta}_i \in [0, 2\pi)$. As a result, we can now determine the corresponding chain code

$$c_i = \left\lfloor \frac{\hat{\theta}_i}{\pi/4} \right\rceil.$$

Clearly, we can see that this procedure splits the unit circle into eight equal parts. Additionally, if the directional change does not exactly align within the eight splits, then the rounding operator $\lfloor\ \rceil$ will approximate the chain code to the nearest integer.

S1.4. *Curvature Chain Code.* Let $\Delta\boldsymbol{c}$ be a $1 \times n$ vector representing the curvature chain code such that each entry is formed from a transformation of the difference between two consecutive chain codes, that is, let $\Delta d_i = c_i - c_{i+1}$ and

$$\Delta c_i = \begin{cases} \Delta d_i - 7 & \Delta d_i > 2, \\ \Delta d_i + 7 & \Delta d_i < 2, \\ \Delta d_i & \text{otherwise.} \end{cases}$$

This simple chain code derivation estimates the curvature and contains information on the convexity of the shape (Wirth, 2004).

S1.5. *Radial Lengths.* Let $\boldsymbol{r}$ be a $1 \times n$ vector of radial lengths, that is, each entry $r_i = \|P_{i\cdot} - P_c\|$, for $i = 1, \ldots, n$, is the Euclidean distance from the boundary point to the shape's centroid. We define the centroid of the polygonal chain $P_c = (x_c, y_c)$ as

$$x_c = \frac{1}{6A} \sum_{i=1}^{n} (x_i + x_{i+1})(x_i y_{i+1} - x_{i+1} y_i)$$

$$y_c = \frac{1}{6A} \sum_{i=1}^{n} (y_i + y_{i+1})(x_i y_{i+1} - x_{i+1} y_i)$$

where

$$A = \frac{1}{2} \sum_{i=1}^{n} (x_i y_{i+1} - x_{i+1} y_i)$$

is the signed area of the shape, obtained using Gauss's area formula. Clearly, the radial lengths are not scale-invariant (as the Euclidean distance is not). Therefore, to properly analyze the structure of $\boldsymbol{r}$, the individual radial lengths must be normalized.

S1.6. *Normalized Radial Lengths.* Let $r_{(n)}$ be the maximum radial length in $\boldsymbol{r}$ such that we can introduce a $1 \times n$ vector of normalized radial lengths, denoted as $\tilde{\boldsymbol{r}}$, where each entry $\tilde{r}_i = r_i \,/\, r_{(n)}$, $i = 1, \ldots, n$. By normalizing the radial lengths, we have obtain a 1-dimensional signal that is scale-invariant and which we can use to analyze the fine details of the shape's contour (Wirth, 2004).

TABLE S1
Components of the output list resulting from the SAFARI procedure.

Component	Description
`desc`	A `data.frame` object of the shape features corresponding to each segmented ROI.
`holes`	An integer matrix containing the holes within each ROI, labeled according to the regions.
`id`	A character vector that is identical to the `id` argument.
`k`	A specified factor to enlarge the polygonal chain by with the default being 3.
`n`	Number of resulting segmented regions.
`plg.chains`	A `list` object where each component is the polygonal chain of a segmented ROI.
`regions`	An integer matrix containing the segmented ROI, labeled from largest to smallest.

*It takes about 0.3 seconds for SAFARI to run a moderate size binary image (300×300 pixels).

TABLE S2
Data notation for shape representations.

Name	Data	Support
Binary Matrix	$\boldsymbol{M}_{W \times L}$	$M_{wl} \in \{0, 1\}$
Polygonal Chain	$\boldsymbol{P}_{(n+1)\times 2}$	$P_{i\cdot} = (x_i, y_i) \in \mathbb{N}^2$
Convex Hull Chain	$\boldsymbol{P}_{\text{CH}}$	$P_{\text{CH}_{i\cdot}} \in \boldsymbol{P}$
Minimum Bounding Box Chain	$\boldsymbol{P}_{\text{MBB}}$	$P_{\text{MBB}_{i\cdot}} \in \mathbb{R}_+^2$
Chain Code	$\boldsymbol{c} = [c_i]_{1\times n}$	$c_i \in \mathbb{N} \cap [0, 7]$
Curvature Chain Code	$\Delta\boldsymbol{c} = [\Delta c_i]_{1\times n}$	$\Delta c_i \in \mathbb{Z} \cap [-2, 2]$
Radial Lengths	$\boldsymbol{r} = [r_i]_{1\times n}$	$r_i \in \mathbb{R}_+$
Normalized Radial Lengths	$\tilde{\boldsymbol{r}} = [\tilde{r}_i]_{1\times n}$	$\tilde{r}_i \in [0, 1]$

Table S3: Overview of the shape features available in the SAFARI package.

Category	Data	Name	Formulae	Range	Interpretation	Invariance: Rotation	Invariance: Scale	Invariance: Translation	Complexity	Reference
		Net Area	$\text{Area}_{net}(\boldsymbol{M}) = \sum_{l,w} M_{wl} = A(\boldsymbol{M})$	$\mathbb{N}$	Number of pixels that make up the region.	•		•	$\mathcal{O}(LW)$	Agu (2014)
	$\boldsymbol{M}$	Thickness[1,2]	$\text{Thickness}(\boldsymbol{M}) = n(\tilde{R})$	$\mathbb{N}$	Number of erosion steps that can be applied to the region before the area equals zero.	•		•	$\mathcal{O}(L^2W^2)$	Wirth (2004)
		Elongation[3]	$\text{Elongation}(\boldsymbol{M}) = \frac{A(\boldsymbol{M})}{2 \cdot \text{Thickness}(\boldsymbol{M})^2}$	$\mathbb{R}_+$	Relationship between the area of the region and the square of its thickness.	•		•	$\mathcal{O}(L^2W^2)$	Wirth (2004)
		Filled Area[4,5]	$\text{Area}_{filled}(\boldsymbol{P}) = \frac{1}{2} \cdot \left\lvert \sum_{i=1}^{n} (x_i y_{i+1} - x_{i+1} y_i) \right\rvert \approx A(\boldsymbol{M})$	$\mathbb{R}_+$	Approximate area of the region.	•		•	$\mathcal{O}(n)$	Agu (2014)
		Perimeter	$\text{Perimeter}(\boldsymbol{P}) = \sum_{i=1}^{n} \lVert P_{i+1\cdot} - P_{i\cdot} \rVert_2 \approx P(\boldsymbol{M})$	$\mathbb{R}_+$	Approximate length of the boundary that makes up the region.	•		•	$\mathcal{O}(n)$	Agu (2014)
	$\boldsymbol{P}$	Circularity	$\text{Circularity}(\boldsymbol{P}) = 4\pi \cdot \text{Area}_{filled}(\boldsymbol{P})\ /\ \text{Perimeter}(\boldsymbol{P})^2$	$[0,1]$	Compactness normalized against a filled circle.	•	•	•	$\mathcal{O}(n)$	Agu (2014)
		Fibre Length	$\text{Fibre}_{length}(\boldsymbol{P}) = \frac{\text{Per}(\boldsymbol{P}) - \sqrt{\text{Per}(\boldsymbol{P})^2 - 16 \cdot \text{Area}_{filled}(\boldsymbol{P})}}{4}$	$\mathbb{R}_+$		•		•	$\mathcal{O}(n)$	Wirth (2004)
		Fibre Width	$\text{Fibre}_{width}(\boldsymbol{P}) = \text{Area}_{filled}(\boldsymbol{P})\ /\ \text{Fibre}_{length}(\boldsymbol{P})$	$\mathbb{R}_+$		•		•	$\mathcal{O}(n)$	Wirth (2004)
		Convex Area	Measurement can be obtained by applying the same formula as in **Filled Area** to $\boldsymbol{P}_{\text{CH}}$.							
Geometric		Convex Perimeter	Measurement can be obtained by applying the same formula as in **Perimeter** to $\boldsymbol{P}_{\text{CH}}$.							
	$\boldsymbol{P}_{\text{CH}}$	Roundness[6]	$\text{Roundness}(\boldsymbol{P}_{\text{CH}}) = 4\pi \cdot \text{Area}_{filled}(\boldsymbol{P})\ /\ \text{Perimeter}(\boldsymbol{P}_{\text{CH}})^2$	$[0,1]$	Relationship between the area of the region and the area of a circle with the same convex perimeter i.e. area-to-perimeter ratio.	•	•	•	$\mathcal{O}(n)$	Wirth (2004)
		Convexity[7]	$\text{Convexity}(\boldsymbol{P}_{\text{CH}}) = \text{Perimeter}(\boldsymbol{P}_{\text{CH}})\ /\ \text{Perimeter}(\boldsymbol{P})$	$[0,1]$	Measures how much the region differs from a convex shape.	•	•	•	$\mathcal{O}(n)$	Wirth (2004)
		Solidity[8]	$\text{Solidity}(\boldsymbol{P}_{\text{CH}}) = \text{Area}_{filled}(\boldsymbol{P})\ /\ \text{Area}_{filled}(\boldsymbol{P}_{\text{CH}})$	$[0,1]$	Measures the density of the region.	•	•	•	$\mathcal{O}(n)$	Wirth (2004)
		Major Axis Length	$\text{Axis}_{length}(\boldsymbol{P}_{\text{MBB}}) = \max_{1 \le i \le 4} \lVert \boldsymbol{b}_i \rVert_2 \ni \boldsymbol{b}_i = P_{\text{MBB}_{i+1\cdot}} - P_{\text{MBB}_{i\cdot}}$	$\mathbb{R}_+$	Length of the region.	•		•	$\mathcal{O}(1)$	
		Major Axis Angle	$\text{Axis}_{angle}(\boldsymbol{P}_{\text{MBB}}) = \arctan\left(\frac{y_j^{MBB}}{x_j^{MBB}}\right) \ni j = \underset{1 \le i \le 4}{\arg\max} \lVert \boldsymbol{b}_i \rVert_2$	$[-\frac{\pi}{2}, \frac{\pi}{2}]$	Orientation of the region.		•	•	$\mathcal{O}(1)$	
	$\boldsymbol{P}_{\text{MBB}}$	Minor Axis Length	$\text{Axis}_{width}(\boldsymbol{P}_{\text{MBB}}) = \min_{1 \le i \le 4} \lVert \boldsymbol{b}_i \rVert_2 \ni \boldsymbol{b}_i = P_{\text{MBB}_{i+1\cdot}} - P_{\text{MBB}_{i\cdot}}$	$\mathbb{R}_+$	Width of the region.	•		•	$\mathcal{O}(1)$	
		Bounding Box Area	Measurement can be obtained by computing $\text{Axis}_{length}(\boldsymbol{P}_{\text{MBB}}) \cdot \text{Axis}_{width}(\boldsymbol{P}_{\text{MBB}})$.							
		Eccentricity	$\text{Eccentricity}(\boldsymbol{P}_{\text{MBB}}) = \text{Axis}_{width}(\boldsymbol{P}_{\text{MBB}})\ /\ \text{Axis}_{length}(\boldsymbol{P}_{\text{MBB}})$	$[0,1]$	Measures the ellipticity of the region.	•	•	•	$\mathcal{O}(1)$	Wirth (2004)
		Curl[9]	$\text{Curl}(\boldsymbol{P}_{\text{MBB}}) = \text{Axis}_{length}(\boldsymbol{P}_{\text{MBB}})\ /\ \text{Fibre}_{length}(\boldsymbol{P})$	$\mathbb{R}_+$	Measures the degree to which a region is "curled up".	•		•	$\mathcal{O}(n)$	Wirth (2004)
	$\Delta\boldsymbol{c}$	Bending Energy	$\text{E}_c(\Delta\boldsymbol{c}) = \frac{1}{n} \sum_{i=1}^{n} (\Delta c_i)^2$	$\mathbb{R}_+$	Energy necessary for a rod to be bent like the region.			•	$\mathcal{O}(n)$	Wirth (2004)
		Total Abs. Curvature[10]	$\kappa_{total}(\Delta\boldsymbol{c}) = \frac{1}{n} \sum_{i=1}^{n} \lvert \Delta c_i \rvert$	$\mathbb{R}_+$	Another measurement for curvature.			•	$\mathcal{O}(n)$	Wirth (2004)
		Radial Mean	$m(\tilde{\boldsymbol{r}}) = \frac{1}{n} \sum_{i=1}^{n} \tilde{r}_i = \bar{r}$	$[0,1]$	Measure macroscopic changes in the boundary of the region.	•	•	•	$\mathcal{O}(n)$	Kilday, Palmieri and Fox (1993)
		Radial S.D.	$\sigma(\tilde{\boldsymbol{r}}) = \sqrt{\frac{1}{n} \sum_{i=1}^{n} (\tilde{r}_i - \bar{r})^2} = s_r$	$[0,1]$	Similar to **Radial Mean** but can also indicate fine boundary changes.	•	•	•	$\mathcal{O}(n)$	Kilday, Palmieri and Fox (1993)
Boundary	$\tilde{\boldsymbol{r}}$	Entropy[11,12]	$\text{E}_r(\tilde{\boldsymbol{r}}) = -\sum_{k=1}^{100} p_k \log(p_k) \ni p_k = P(\omega < \tilde{\boldsymbol{r}} < \omega + \Delta)$	$\mathbb{R}_+$	Probabilistic measure of how well the radial lengths can be estimated.	•	•	•	$\mathcal{O}(n)$	Kilday, Palmieri and Fox (1993)
		Area Ratio[13]	$\text{Area}_{Ratio}(\tilde{\boldsymbol{r}}) = \frac{1}{n\bar{r}} \sum_{i=1}^{n} (\tilde{r}_i - \bar{r}) \cdot \text{I}(\tilde{r}_i > \bar{r})$	$[0,1]$	Measures the macroscopic characteristics of the region.	•	•	•	$\mathcal{O}(n)$	Kilday, Palmieri and Fox (1993)
		Zero Crossing Count[14]	$\text{Zero}_{Crossing}(\tilde{\boldsymbol{r}}) = \sum_{i=1}^{n} \text{I}\left\{(\tilde{r}_i - \bar{r}) \cdot (\tilde{r}_{i+1} - \bar{r}) < 0\right\}$	$\mathbb{N}$	Captures the fine detail of the boundary.	•	•	•	$\mathcal{O}(n)$	Kilday, Palmieri and Fox (1993)
		Normalized Moment Classifier[15]	$\text{Nm}(\tilde{\boldsymbol{r}}) = \left\{ \left[\frac{1}{n} \sum_{i=1}^{n} (\tilde{r}_i - \bar{r})^4\right]^{1/4} - \left[\frac{1}{n} \sum_{i=1}^{n} (\tilde{r}_i - \bar{r})^2\right]^{1/2} \right\} \Big/ \ \bar{r}$	$[0,1]$	Measures the roughness of the region, based on the moments of inertia of $\tilde{\boldsymbol{r}}$.	•	•	•	$\mathcal{O}(n)$	Pohlman et al. (1996)
Topological	$\boldsymbol{M}$	Number of Holes[16]	$\text{N}_H(\boldsymbol{M}) = \lVert f_S(\boldsymbol{H}) \rVert_{\max} \ni \boldsymbol{H} = f_H(\boldsymbol{M}) - \boldsymbol{M}$	$\mathbb{N}$		•	•	•	$\mathcal{O}(L^2W^2)$	
		Number of Protrusions	$\text{N}_P(\boldsymbol{M}) = A(\tilde{\boldsymbol{R}}) \ni \tilde{\boldsymbol{R}} = f_H(\boldsymbol{M}) - \hat{\boldsymbol{R}}, \hat{\boldsymbol{R}} = (f_H(\boldsymbol{M}) \ominus \boldsymbol{E}) \circ \boldsymbol{E}$	$\mathbb{N}$				•	$\mathcal{O}(L^2W^2)$	

1 We note that $\tilde{R} = \{\boldsymbol{M}_i \mid i = 1, \ldots, n, \boldsymbol{M}_i = \boldsymbol{M}_{i-1} \ominus \boldsymbol{E}, \boldsymbol{M}_0 = \boldsymbol{M}, A(\boldsymbol{M}_n) = 0\}$ is the set containing the repeated erosion steps applied to the region, where $\boldsymbol{E}$ is a structuring element.
2 The holes within the region must be filled before computing the measurement.
3 This measurement applies to curved regions, unlike eccentricity.
4 Estimates measurement using Gauss' area formula for polygons (Braden, 1986).
5 Approximation worsens as the true size of the region increases.
6 This measurement excludes local irregularities which makes it relatively insensitive to irregular boundaries.
7 This measurement accounts for irregularities, such as an irregular boundary.
8 A measure less than one accounts for a region with an irregular boundary or containing holes.
9 As the measurement decreases, the degree to which the region is "curled up" increases.
10 The minimum value is obtained for a convex shape.
11 Relies on the 100-bin histogram of the radial lengths where p_k is the kth entry and $\omega = k\ /\ 100, \Delta = 0.01$.
12 Also incorporates the roundness and roughness of the region.
13 This measurement can be simplified as how much of the region is outside the circle with a radius defined by $\bar{r}$.
14 This measurement can be simplified as the number of times the region crosses the contour of the circle with a radius defined by $\bar{r}$.
15 A rough boundary will have a greater normalized moment classifier than a smooth boundary.
16 We define $f_H : \boldsymbol{X} \to \boldsymbol{Y}$ and $f_S : \boldsymbol{X} \to \boldsymbol{Y}$, where $\boldsymbol{X}, \boldsymbol{Y}$ are binary matrices, as the procedures to fill the holes within a region and segment the regions within an image, respectively (Gonzalez, Woods and Eddins, 2020).

TABLE S4

Patient characteristics of the National Lung Screening Trial (NLST) and The Cancer Genome Atlas (TCGA) datasets. Values are either mean ± standard deviation, or number (percentage). In the case of the survival time, we use the median instead of the mean.

	NLST	TCGA
Number of Patients	143	61
Age (in years)	64.01 ± 5.19	58.26 ± 12.48
Survival Time (in days)	1517 ± 730.04	403.93 ± 291.52
Karnofsky Score (0-100)	–	81.15 ± 12.92
**Status**		
Alive	98 (68.53%)	21 (34.42%)
Dead	45 (31.47%)	40 (65.57%)
**Gender**		
Male	80 (55.94%)	42 (68.85%)
Female	63 (44.06%)	19 (31.15%)
**Smoking Status**		
Yes	75 (52.45%)	–
No	68 (47.55%)	–
**Cancer Stage**		
Stage I	95 (66.43%)	–
Stage II	15 (10.49%)	–
Stage III	23 (16.08%)	–
Stage IV	10 (6.99%)	–

TABLE S5

Univariate analysis of individual shape features in the National Lung Screening Trial (NLST) dataset. A Cox proportional-hazards (CoxPH) model was fitted to each centered and scaled feature, clustered to adjust for patients with multiple samples.

	Coefficients	Hazard Ratio (HR)	Standard Error (SE)	Robust Standard Error	p-value*
Net Area	0.2679	1.3072	0.0864	0.1173	**0.0224**
Thickness	0.2733	1.3143	0.0943	0.1119	**0.0146**
Elongation	-0.1467	0.8636	0.1153	0.1035	0.1563
Area Filled	0.2675	1.3066	0.0869	0.1126	**0.0175**
Perimeter	0.2900	1.3364	0.1026	0.1280	**0.0235**
Circularity	0.1623	1.1762	0.1066	0.1163	0.1627
Convex Area	0.2853	1.3301	0.0888	0.1182	**0.0158**
Convex Perimeter	0.3467	1.4144	0.1017	0.1331	**0.0092**
Roundness	0.1249	1.1331	0.1140	0.1268	0.3243
Convexity	0.1165	1.1236	0.1154	0.1359	0.3913
Solidity	0.2796	1.3226	0.1210	0.1383	**0.0433**
Major Axis Length	0.3934	1.4820	0.1046	0.1359	**0.0038**
Major Axis Angle	-0.0213	0.9790	0.1114	0.1166	0.8553
Minor Axis Length	0.2857	1.3307	0.1020	0.1206	**0.0179**
Bounding Box Area	0.3092	1.3624	0.0908	0.1175	**0.0085**
Eccentricity	-0.1456	0.8645	0.1101	0.1239	0.2398
Fibre Length	0.3093	1.3625	0.0952	0.1132	**0.0063**
Fibre Width	0.2860	1.3310	0.1028	0.1281	**0.0255**
Curl	-0.0705	0.9319	0.1160	0.1222	0.5640
Bending Energy	0.0054	1.0054	0.1074	0.1010	0.9572
Total Abs. Curvature	0.0074	1.0074	0.1072	0.0991	0.9405
Radial Mean	0.0682	1.0706	0.1107	0.1271	0.5916
Radial S.D.	-0.0586	0.9431	0.1079	0.1052	0.5776
Entropy	-0.0624	0.9395	0.1060	0.1037	0.5475
Area Ratio	-0.0504	0.9509	0.1094	0.1131	0.6560
Zero Crossing	-0.0354	0.9652	0.1087	0.1074	0.7418
Normalized Moment	-0.1409	0.8686	0.1152	0.1383	0.3082
Number of Holes	0.2289	1.2572	0.0844	0.0911	**0.0120**
Number of Protrusions	0.2877	1.3334	0.1029	0.1282	**0.0248**

*Bolding signifies features with p-value ≤ 0.05

Table S6

Comparison of the results obtained in our univariate study, compared to those in Wang et al. (2018). The p-value in Wang et al. (2018) corresponds to either the sum of the shape feature for all regions or the shape feature for the main region, whichever was more significant. Additionally, bolding signifies features with p-value $\leq$ 0.05 and we show features not present in either study.

	Our Study	Wang's Paper
Average Tumor Probability	–	0.78
Net Area	**0.0224**	**0.0033**
Thickness	**0.0146**	–
Elongation	0.1563	–
Area Filled	**0.0175**	**0.0029**
Perimeter	**0.0235**	**0.0034**
Circularity	0.1627	**0.019***
Convex Area	**0.0158**	**0.0047**
Convex Perimeter	**0.0092**	–
Roundness	0.3243	–
Convexity	0.3913	–
Solidity	**0.0433**	0.16
Major Axis Length	**0.0038**	**0.0099**
Major Axis Angle	0.8553	0.92
Minor Axis Length	**0.0179**	**0.030**
Bounding Box Area	**0.0085**	–
Eccentricity	0.2398	0.13
Extent	–	0.34
Fibre Length	**0.0063**	–
Fibre Width	**0.0255**	–
Curl	0.5640	–
Bending Energy	0.9572	–
Total Abs. Curvature	0.9405	–
Radial Mean	0.5916	–
Radial S.D.	0.5776	–
Entropy	0.5475	–
Area Ratio	0.6560	–
Zero Crossing	0.7418	–
Normalized Moment	0.3082	–
Number of Holes	**0.0120**	**0.0033**
Number of Protrusions	**0.0248**	–
Number of Regions	–	0.48

*Measure corresponds to a variation of the formula we used.

Table S7

Univariate analysis of individual shape features in The Cancer Genome Atlas (TCGA) dataset. A Cox proportional-hazards (CoxPH) model was fitted to each centered and scaled feature.

	Coefficients	Hazard Ratio (HR)	Standard Error (SE)	p-value
Net Area	0.5784	1.7832	0.1706	**<0.001**
Thickness	0.327	1.3868	0.1821	0.0726
Elongation	-0.1706	0.8431	0.287	0.5522
Area Filled	0.4361	1.5467	0.1843	**0.0180**
Perimeter	0.4182	1.5192	0.1975	**0.0342**
Circularity	0.0962	1.101	0.1631	0.5555
Convex Area	0.555	1.742	0.1936	**0.0041**
Convex Perimeter	0.4917	1.6351	0.2001	**0.0140**
Roundness	0.2225	1.2492	0.1839	0.2264
Convexity	0.099	1.1041	0.1743	0.5700
Solidity	0.1957	1.2161	0.1807	0.2790
Major Axis Length	0.4776	1.6121	0.2006	**0.0173**
Major Axis Angle	0.5612	1.7528	0.2104	**0.0076**
Minor Axis Length	0.5108	1.6666	0.198	**0.0099**
Bounding Box Area	0.5371	1.7111	0.1913	**0.005**
Eccentricity	0.0695	1.072	0.1501	0.6435
Fibre Length	0.2615	1.2989	0.1776	0.1410
Fibre Width	0.3954	1.485	0.1966	**0.0443**
Curl	-0.0652	0.9368	0.2386	0.7845
Bending Energy	0.14	1.1502	0.165	0.3964
Total Abs Curvature	0.1501	1.1619	0.1655	0.3646
Radial Mean	0.2061	1.2289	0.171	0.2281
Radial Sd	0.0786	1.0818	0.1649	0.6336
Entropy	0.146	1.1572	0.1624	0.3689
Area Ratio	0.0869	1.0908	0.1696	0.6084
Zero Crossing	0.2271	1.255	0.1638	0.1656
Normalized Moment	0.2168	1.2421	0.1712	0.2053
Number Holes	0.4667	1.5947	0.1963	**0.0174**
Number Protrusions	0.3618	1.4359	0.1926	0.0603

*Bolding signifies features with p-value ≤ 0.05

Table S8: A summary of SAFARI and other related shape analysis tools in R.

Tool	Description/Objectives	Data	# of Shape Features	Applications	Latest Version	Availability			Citation
						Article	Package	Web Tool	
EBImage	This package provides general purpose functionality for image processing and analysis, by offering tools to segment cells and extract quantitative cellular descriptors and automating image processing tasks.	$\boldsymbol{M}$	9	Microscopy-based cellular assays	4.36.0 (2021-12-19)	✓	✓		[1]
SAFARI	This package provides functionality for image processing and shape analysis in the context of segmented medical images generated by deep learning-based methods or standard image processing algorithms and produced from different medical imaging types. Specifically, offers tools to segment regions of interest and extract quantitative shape descriptors.	$\boldsymbol{M}, \boldsymbol{P}, \boldsymbol{c}, \boldsymbol{r}$	29	AI-segmented images	0.1.0 (2021-02-25)	✓	✓	✓	[2]
shapes	This package offers routines for the statistical analysis of landmark shapes.	$\boldsymbol{P}$	0	Landmark shapes	1.2.6 (2021-03-30)	✓	✓		[3]
wvtool	This package intendeds to facilitate preprocessing and analyzing wood images toward automated recognition.	$\boldsymbol{M}$	0	Wood images	1.0.0 (2016-11-08)		✓		[4]

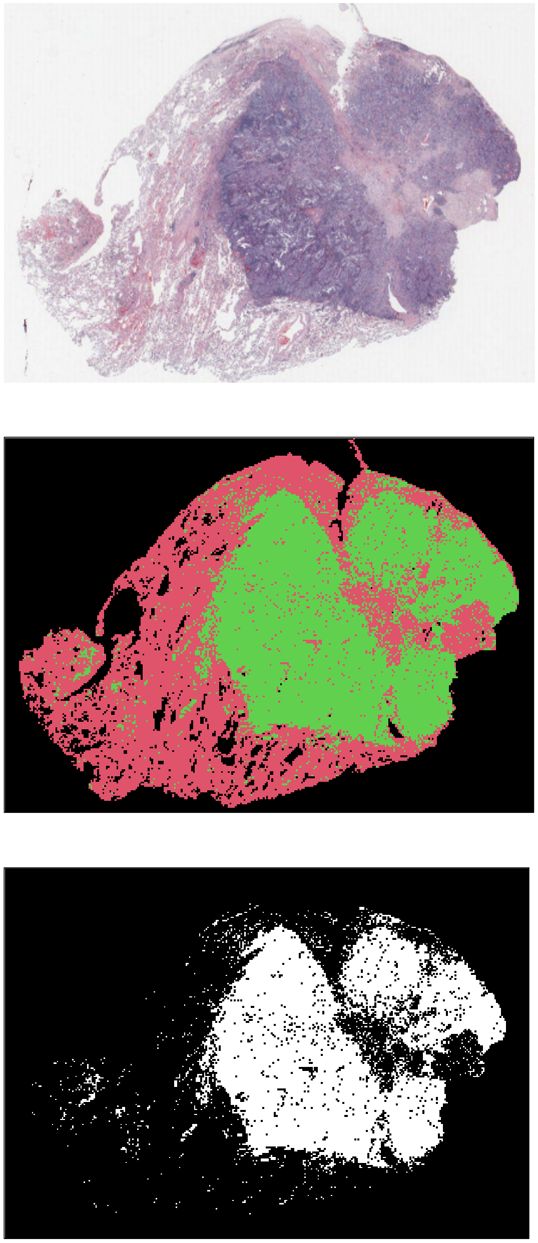

Fig S1: An example of a whole-slide image from the National Lung Screening Trial (NLST) cohort processed by an Automated Tumor Recognition System Wang et al. (2018) and then converted into a binary format. The images are whole-slide pathology image (top), segmented three-class image (middle), and segmented two-class or binary image (bottom).

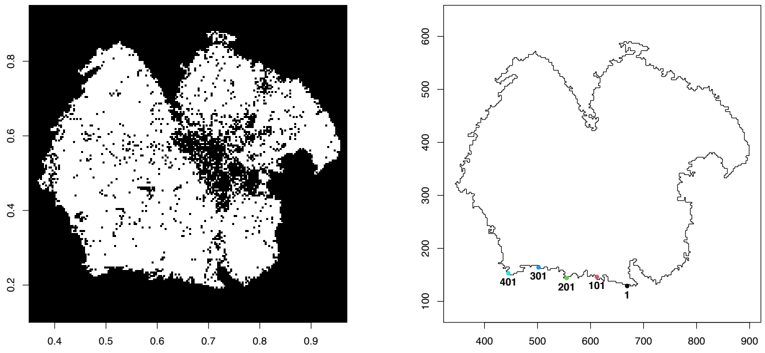

Fig S2: An example of the binary matrix (left) and its corresponding polygonal chain (right). The polygonal chain also shows the starting point and four sample points to demonstrated the contour's clockwise direction.

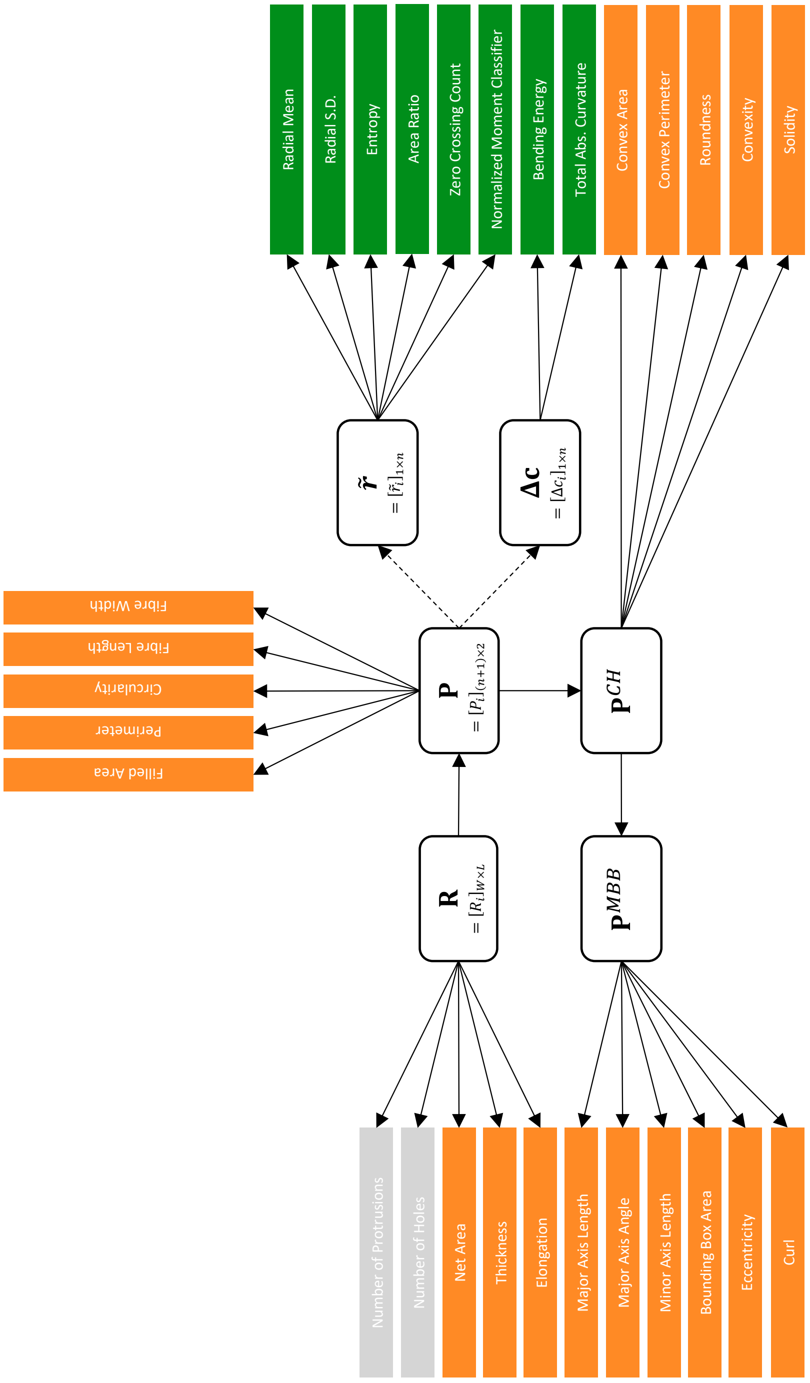

Fig S3: Dependencies of shape features and representations. Colored boxes refer to the shape feature categories. Orange denotes geometric, green boundary, and grey topological.

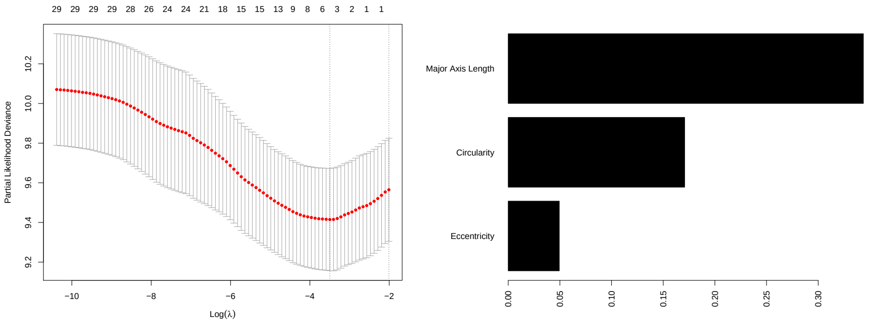

Fig S4: Results from the regularized Cox proportional-hazards (CoxPH) model in **Downstream Analysis II: Predictive Performance**. The left figure shows the mean cross-validated errors, based on the Partial Likelihood Deviance, for each tuning parameter. The right figure shows the importance of each feature kept by the regularized Cox model, based on the magnitude of each coefficient.

REFERENCES

Agu, E. (2014). Digital Image Processing (CS/ECE 545) Lecture 8: Regions in Binary Images (Part 2) and Color (Part 1). Accessed: May 13, 2021.

Braden, B. (1986). The Surveyor's Area Formula. *The College Mathematics Journal* **17** 326–337.

Gonzalez, R. C., Woods, R. E. and Eddins, S. L. (2020). *Digital image processing using MATLAB*, Third edition ed. Gatesmark Publishing, Knoxville.

Kilday, J., Palmieri, F. and Fox, M. D. (1993). Classifying mammographic lesions using computerized image analysis. *IEEE Transactions on Medical Imaging* **12** 664–669.

Pohlman, S., Powell, K. A., Obuchowski, N. A., Chilcote, W. A. and Grundfest-Broniatowski, S. (1996). Quantitative classification of breast tumors in digitized mammograms. *Medical Physics* **23** 1337–1345.

Wang, S., Chen, A., Yang, L., Cai, L., Xie, Y., Fujimoto, J., Gazdar, A. and Xiao, G. (2018). Comprehensive analysis of lung cancer pathology images to discover tumor shape and boundary features that predict survival outcome. *Scientific Reports* **8** 10393.

Wirth, M. A. (2004). Shape Analysis & Measurement. Accessed: May 13, 2021.
